# Dr. Antonio Savaresi, One of the Physicians to the French Army in Ægypt, on the Cure and Prevention of the Endemic Ophthalmia of That Country

**Published:** 1801-10

**Authors:** G. Blane

**Affiliations:** Sackville Street


### *To the Editors of the Medical and Phyſical Journal.*

Gentlemen,

Sir SIDNEY SMITH ſent me lately a ſmall Treatiſe on the Egyptian Blindneſs. I have extracted the moſt intereſting part of it, which Sir Sidney recommended to me to tranſlate, and ſend to Egypt for the benefit of our army.

Will you have the goodneſs to inſert it in your next Journal, and ſend me a few copies to ſend to Egypt.

I am, &c.

*Sackville Street,*
*September* 24, 1801.

G. BLANE.

---

Dr. Antonio Savaresi, *one of the Phyſicians to the French Army in Ægypt, on the Cure and Prevention of the endemic Ophthalmia of that Country. Tranſlated from the Italian. Communicated by* Dr. Blane.

He firſt divides this complaint into the ſthenic and aſthenic; the one depending on an exceſs, the other on a defect of tone. The former affects the bulb of the eye; the latter, ſometimes the *tarſus*, ſometimes the *tunica conjunctiva*.

In the beginning I purge in all the three ſpecies, without diſtinction, with an ounce of magneſia vitriolata, otherwiſe called Epſom ſalts.

The ſthenic ophthalmia requires very cloſe and ſtrict attention, inaſmuch as the cure depends on the efficiency of the firſt remedies. In this caſe, a bliſter to the nape of the neck, and local bleeding from the temporal or jugular vein, are of great utility, and ought not to be omitted. An hour after the letting of blood, a ſenſible change in the complaint is perceived, and next day the violent pain of the part and the ſevere headache diminiſh, or at leaſt ceaſe to torture the patient. This effect is often retarded, and the complaint advances, accompanied panied with a flight feverifhnefs. In order to ftop this, it is neceffary to repeat the bleeding and the purges.

A low diet is prefcribed, a decoction of barley with cream of tartar, and a refolving collyrium, compofed of opium diffolved in fpirit of wine, and in decoction of faffron, which contributes to calm.

This method of cure fhould be continued till the fwelling of the eyes is diminifhed, and the eye-lids begin to be turned up with a degree of fwelling, an appearance which always proceeds from the weakening and relaxation of the veffels. In confequence of this change a faponaceous collyrium is ordered, which confifts in a folution of foap in fpirit of wine, by the ufe of which the eye-lids refume their natural fituation, and eafily open, fo that the cornea being now vifible is found fometimes red or covered with fpots. In the firft cafe, cold water with vinegar is employed with good fuccefs; and in the fecond, recourfe is had to the dry collyrium, compofed of fugar-candy, alum, and nitre, which deftroys the fpots in a few days. By means of thefe topical and internal remedies, a compleat cure is effected in the fpace of a month or two. If the complaint fhould not be removed in that time, there will be too much reafon to defpair of a cure.

With refpect to the cure of the fecond fpecies of ophthalmia, I have applied only a tonic collyrium of white vitriol diffolved in vinegar, water, and proof fpirit. This remedy has afforded the utmoft relief, and has cured the complaint radically in twenty days or a month.

Another collyrium made of common falt diffolved in vinegar and water, has ferved to cure the third fpecies of ophthalmic inflammation, which is more fimple, but obftinate like the preceding. In the maritime countries of Italy, I have feen this indifpofition cured with baths of fea water.

Many praife the application of emollient and refolving cataplafms in all the three fpecies. Obfervation teaches us that this remedy is noxious, fince it relaxes the part, increafes the pain, and produces other evils.

Such has been the curative method which I have ufed in the military hofpitals of Ægypt, to fave from blindnefs thofe unfortunate foldiers who were attacked with this difeafe, and placed under my care. Of a thoufand, or thereabouts, who were affected with this diforder, I had the mortification to fee two entirely lofe their fight, and fome others lofe the fight of one eye.

### Preventive Means.

The means I am going to point out, cannot be practifed by foldiers, as their neceffary duties do not admit of it; but they may may be of aſſiſtance to thoſe who have the proper convenience for availing themſelves of them.

Firſt, expoſure to the rays of the ſun with the head uncovered ſhould be avoided, and to the humidity of the night without ſome precaution to ſhelter oneſelf from it. In the ſecond plan, it is neceſſary to bathe the eyes twice or thrice a day with fair water mixed with vinegar or lemon juice, eſpecially when the organ has been irritated by duſt, ſmoak, or any external accident; and when it has been weakened by too much ſunſhine or humidity, it ought to be ſprinkled with ſpirituous or other tonic liquors. Finally, it will be adviſeable to abſtain from ſalted food, and at the ſame time to maintain ſuitable perſpiration; to preſerve the hair of the head a little long, to avoid expoſure to cold after being heated, and to attend to the inteſtinal evacuations.

The ſucceſs of theſe ſimple preſervatives has been confirmed by obſervations and experience. When ſeaſonably practiced, they prevent the diſorder, and preſerve the ſight.

